# Failure of Implantable Cardioverter-defibrillator Pacing Capture in BRASH Syndrome with Severe Hyperkalemia

**DOI:** 10.19102/icrm.2026.17071

**Published:** 2026-07-15

**Authors:** Stephen Roche, Michael Kanan, Deana Mikhalkova

**Affiliations:** 1 Department of Internal Medicine, Saint Louis University School of Medicine, St. Louis, MO, USA; 2 Department of Cardiology, SSM Health Saint Louis University Hospital, St. Louis, MO, USA

**Keywords:** BRASH syndrome, hyperkalemia, implantable cardioverter-defibrillator, renal failure

## Abstract

This case report describes a 54-year-old woman with end-stage renal disease and heart failure who presented with BRASH syndrome: a synergistic cycle of bradycardia, renal failure, atrioventricular nodal blockade, shock, and hyperkalemia. The patient, who was adherent to metoprolol and amiodarone, presented with a profound ventricular escape rhythm of 16 bpm and hemodynamic instability after missing 4 days of hemodialysis. An interesting complication was the failure of her implantable cardioverter-defibrillator (ICD) to achieve pacing capture, a phenomenon likely driven by a potassium level of 7.8 mEq/L causing myocardial inexcitability. Additionally, the recent uptitration of a carotid sinus nerve stimulator for baroreflex activation therapy may have contributed to the bradycardic cycle. Immediate medical management of hyperkalemia with calcium, insulin, and dextrose successfully restored ICD capture, followed by emergent dialysis, which normalized her rhythm. This case emphasizes that BRASH syndrome should be considered in undifferentiated bradycardia, particularly when refractory to atropine, and highlights that correcting the underlying metabolic derangement is essential to restoring cardiac excitability and device function.

## Introduction

Bradycardia–renal failure–atrioventricular nodal blockade–shock–hyperkalemia (BRASH) syndrome describes a cascading effect of acute kidney injury or missed dialysis, whereby the acute insult results in hyperkalemia and impaired clearance of atrioventricular (AV) nodal–blocking agents, resulting in bradycardia and shock.^1–6^ BRASH syndrome provides a unique challenge for clinicians, as the constellation of symptoms is often refractory to routine management methods such as atropine; rapid identification of the syndrome and prompt treatment of the underlying pathophysiology are necessary.^1,6^

We present a case of BRASH syndrome with severe hyperkalemia in a patient taking amiodarone and metoprolol, brought on by missed dialysis and baroreflex activation therapy, complicated by failure of implantable cardioverter-defibrillator (ICD) pacing capture.

## Case presentation

A 54-year-old woman with a past medical history of end-stage renal disease on hemodialysis, heart failure with reduced ejection fraction (23%) with an ICD, severe tricuspid regurgitation, and paroxysmal atrial fibrillation status post direct-current cardioversion presented to the emergency department complaining of nausea, dizziness, and fatigue. Her baseline medication regimen, to which she was adherent, included 25 mg of metoprolol succinate twice daily and 200 mg of amiodarone once daily. She recently had a carotid sinus nerve stimulator (CSNS) placed, and 2 days prior, it was uptitrated from 1 to 3 mA.

In the emergency department, her heart rate was 16 bpm, with unclear blood pressure readings ranging from 35/20 to 112/92 mmHg. A 12-lead electrocardiogram (ECG) was recorded **(Figure 1)**, on which non-captured ICD pacing spikes were seen, with a ventricular rate of 16 bpm in a ventricular escape rhythm. On telemetry, there was no intermittent ventricular capture. It was suspected that the ICD was malfunctioning. One dose of 0.5 mg of intravenous (IV) atropine was administered without improvement in heart rate. Device interrogation was initiated, and a sheath introducer was placed with plans for transvenous pacemaker (TVP) placement. Before the TVP was placed, laboratory workup revealed a potassium level of 7.8 mEq/L. The patient was immediately given 2 g of IV calcium gluconate, 5 units of regular insulin, and 25 g of IV dextrose. Within several minutes, her heart rate increased to 50 bpm, the rate at which her ICD was pacing. Telemetry at this time demonstrated a wide-complex paced ventricular rhythm at 50 bpm, indicating that the ICD was now capturing. ICD interrogation revealed an appropriately functioning device, and the decision was made to not place a TVP. Her CSNS was temporarily disabled with a donut magnet given concerns that the recent increase in stimulation current may have been contributory to her bradycardia. Emergent dialysis was initiated, reducing her serum potassium level to 3.8 mEq/L. A repeat ECG was obtained, as shown in **Figure 2**. At this time, she had returned to a short P–R sinus rhythm with a rate in the 70s, not requiring pacing.

**Figure 1:**
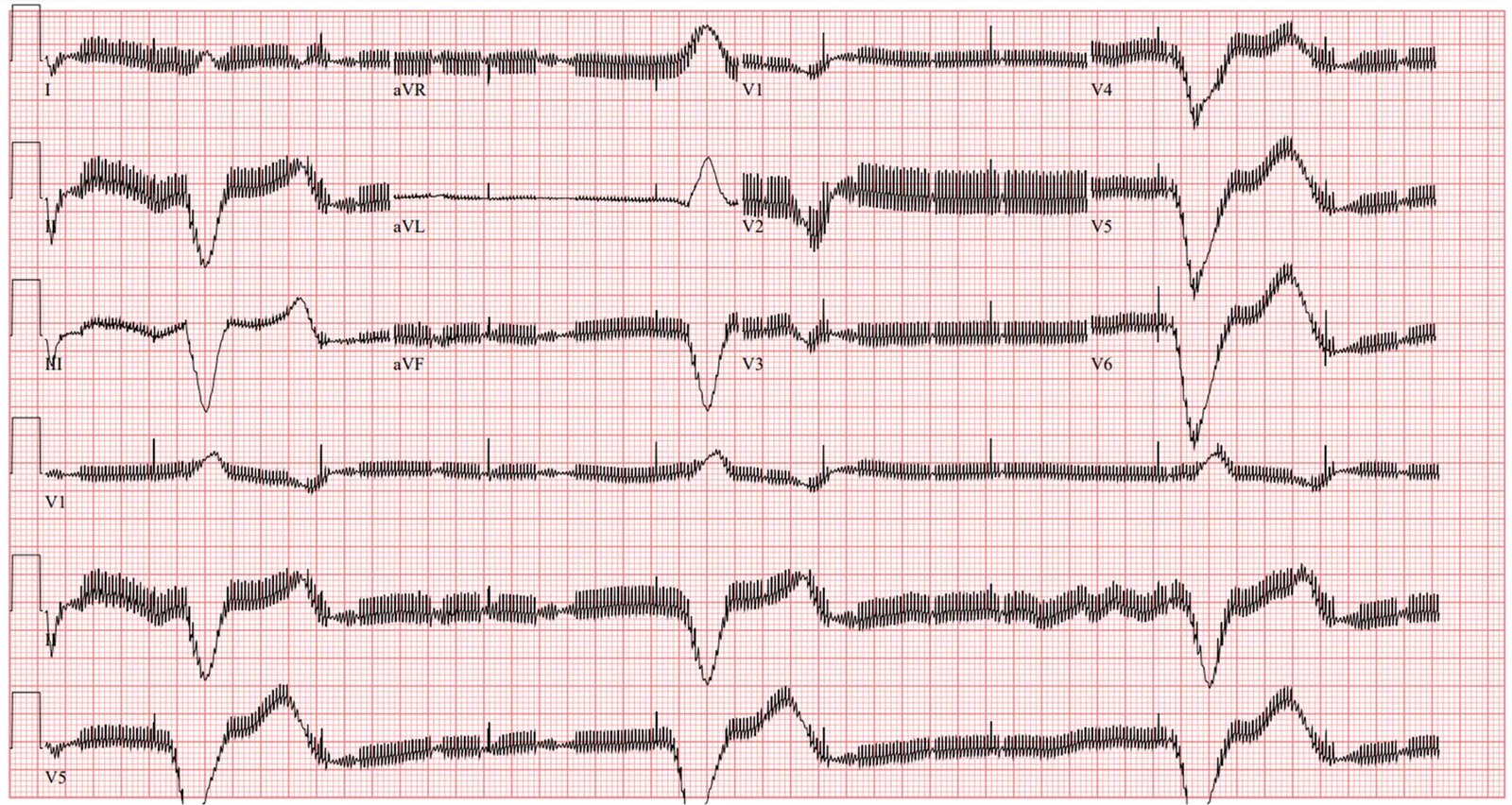
Twelve-lead electrocardiogram performed on arrival. The ventricular rate is 16 bpm in a ventricular escape rhythm with non-captured implantable cardioverter-defibrillator pacing spikes at a rate of 50 bpm. Carotid sinus nerve stimulator artifact is observed. This is consistent with a nonspecific intraventricular conduction block.

**Figure 2:**
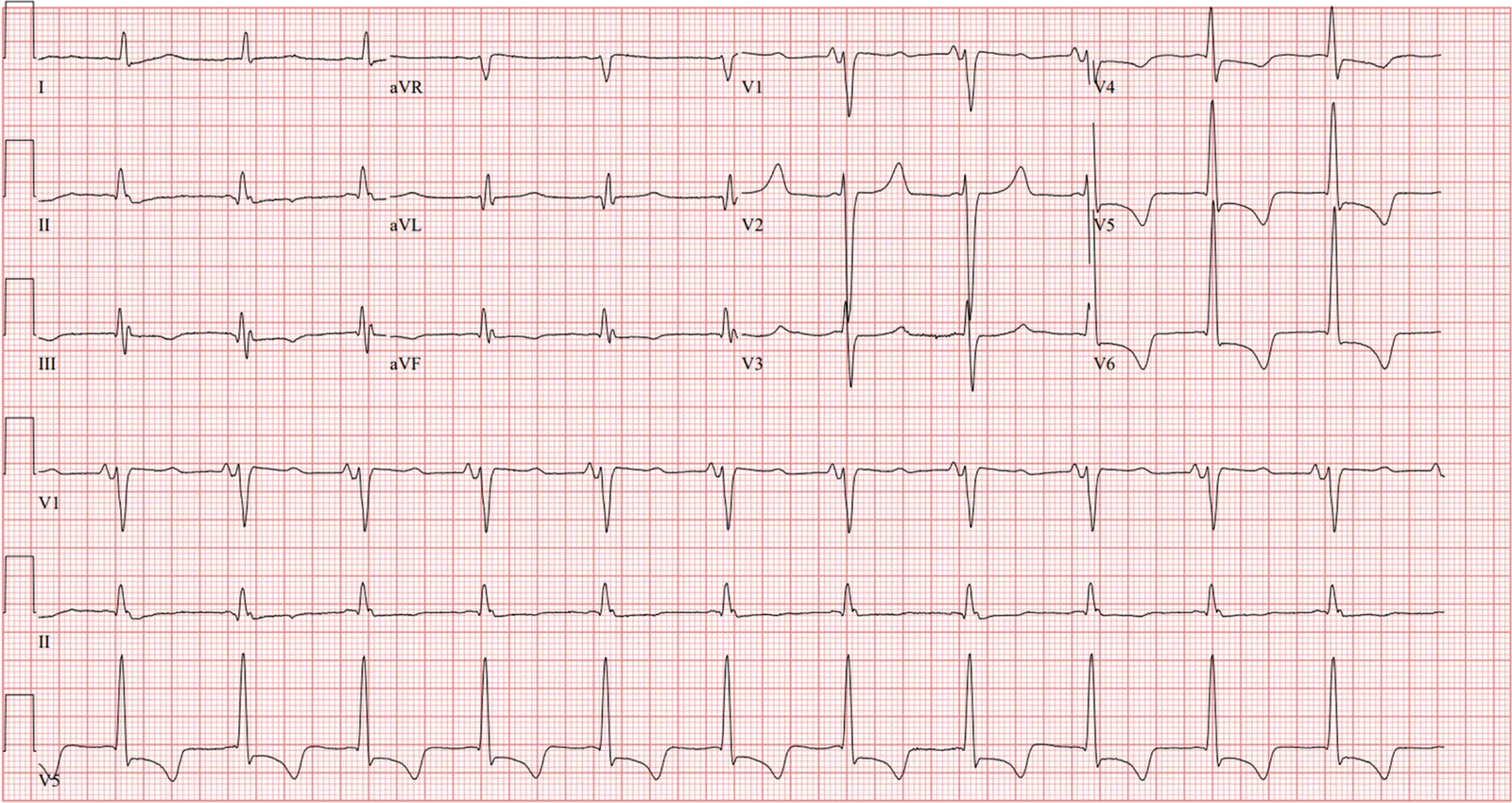
Twelve-lead electrocardiogram after dialysis, with an interval pause of the carotid sinus nerve stimulator. The rhythm has converted to a sinus rhythm with short P–R interval, no longer requiring implantable cardioverter-defibrillator activity. Left ventricular hypertrophy is also demonstrated, with QRS widening and repolarization.

It was later found that she had not received her routine hemodialysis for 4 days prior to admission. The remainder of her hospital course was uncomplicated, and she was discharged home with a reduced dose of oral amiodarone of 100 mg daily.

Saint Louis University’s institutional review board (IRB) was not consulted, as it does not require patient consent or explicit IRB approval for case studies in which the patient cannot be identified from the provided material.

## Discussion

We present a case of severe bradycardia and failure of ICD pacing capture in the setting of BRASH syndrome.

BRASH syndrome is characterized by a synergistic feedback loop rather than a linear progression.^1,4^ In this patient, the cycle likely began with missed dialysis, leading to acute-on-chronic kidney injury and hyperkalemia. This hyperkalemia,^7–10^ combined with her baseline AV nodal–blocking agents (metoprolol^1^ and amiodarone^11^), induced profound bradycardia. Given her severely reduced ejection fraction of 23%, the resulting bradycardia significantly compromised cardiac output and renal perfusion, thus worsening the patient’s kidney injury and perpetuating the cycle. The feedback loop was broken by the management of hyperkalemia, both medically and with emergent dialysis.

An interesting feature of this case is the potential contribution from the CSNS. The device was uptitrated from 1 to 3 mA 2 days prior to presentation. Baroreflex activation therapy has been shown to be effective in heart failure by reducing sympathetic and increasing parasympathetic signaling to the heart.^12^ This mechanism, by decreasing heart rate,^13^ can theoretically contribute to the BRASH cycle by further worsening the cardiac output and acute kidney injury. Though the missed dialysis was likely the primary inciting factor in this patient, the recent CSNS current increase may have played a role in her bradycardia, and promptly disabling it was appropriate management.

While a combination of hyperkalemia, AV nodal blockers, and baroreflex activation therapy uptitration contributed to our patient’s presentation, the failure of ICD pacing capture was likely primarily driven by the hyperkalemia. Several cases of hyperkalemia-induced failure of pacemaker/ICD capture have been reported in the literature.^14–19^ The proposed mechanism includes an increase in the resting cardiomyocyte membrane potential, resulting in relative inactivation of voltage-gated sodium channels and subsequent failure of depolarization despite pacing spikes.^14^ In this case, the return of pacing at the programmed rate of 50 bpm after the administration of calcium, insulin, and dextrose supports hyperkalemia as a primary contributor to the failure to capture in this case.

Finally, this case provides some educational insights into the management of BRASH syndrome. First, the failure of atropine to improve the heart rate is predictable, as the cause of bradycardia was myocardial inexcitability due to hyperkalemia, rather than inadequate impulse formation. In the undifferentiated bradycardic patient, this treatment failure can hint at the underlying pathophysiology. Prompt recognition of the cause is essential for appropriate management. In this case, recognition of the disorder enabled the medical team to avoid the risks of an unnecessary TVP placement.

In addition, the variability in the patient’s initial blood pressure readings highlights a limitation of automated monitoring. On arrival, the patient’s blood pressure readings ranged from 30s/20s to 110s/90s. While BRASH syndrome causes hemodynamic instability, these recordings likely represent measurement artifact. Oscillometric blood pressure cuffs can have significant error in severe arrhythmia^20^; manual blood pressure measurement or arterial line placement is more reliable.

## Conclusions

We report a case of ICD pacing capture failure in BRASH syndrome, which demonstrates rapid ECG normalization as appropriate management is initiated. BRASH syndrome is an essential diagnosis to consider in patients with symptomatic bradycardia, particularly those resistant to treatment with atropine. Diagnostic testing, including a metabolic panel and 12-lead ECG, can assist in the diagnosis. Early treatment aimed at correction of the underlying pathophysiology—including the management of hyperkalemia and kidney injury—is essential to break the cycle. This case also demonstrates that, in addition to AV nodal blockers, baroreflex activation therapy may play a role in the bradycardia seen in BRASH syndrome.
